# The Influence of Electrolytes on the Performance of Self-Powered Photoelectrochemical Photodetector Based on *α*-Ga_2_O_3_ Nanorods

**DOI:** 10.3390/ma17153665

**Published:** 2024-07-25

**Authors:** Junjie He, Chenyang Tao, Yanan Zhang, Jiufu Sun, Xiangyun Zhang, Shujie Jiao, Dongbo Wang, Jinzhong Wang

**Affiliations:** School of Materials Science and Engineering, Harbin Institute of Technology, Harbin 150001, China; a1097448132@163.com (J.H.); taochenyanghit@163.com (C.T.); 18846077725@163.com (Y.Z.); 2021112182@stu.hit.edu.cn (J.S.); 2021111921@stu.hit.edu.cn (X.Z.); wangdongbo@hit.edu.cn (D.W.); jinzhong_wang@hit.edu.cn (J.W.)

**Keywords:** α-Ga_2_O_3_, self-powered photodetector, photoelectrochemical detector, electrolyte

## Abstract

Photodetectors have a wide range of applications across various fields. Self-powered photodetectors that do not require external energy have garnered significant attention. The photoelectrochemical type of photodetector is a self-powered device that is both simple to fabricate and offers high performance. However, developing photoelectrochemical photodetectors with superior quality and performance remains a significant challenge. The electrolyte, which is a key component in these detectors, must maintain extensive contact with the semiconductor without degrading its material quality and efficiently catalyze the redox reactions of photogenerated electrons and holes, while also facilitating rapid charge carrier transport. In this study, α-Ga_2_O_3_ nanorod arrays were synthesized via a cost-effective hydrothermal method to achieve a self-powered solar-blind photodetector. The impacts of different electrolytes—Na_2_SO_4_, NaOH, and Na_2_CO_3_—on the photodetector was investigated. Ultimately, a self-powered photodetector with Na_2_SO_4_ as the electrolyte demonstrated a stable photoresponse, with the maximum responsivity of 0.2 mA/W at 262 nm with the light intensity of 3.0 mW/cm^2^, and it exhibited rise and decay times of 0.16 s and 0.10 s, respectively. The α-Ga_2_O_3_ nanorod arrays and Na_2_SO_4_ electrolyte provided a rapid pathway for the transport of photogenerated carriers and the built-in electric field at the semiconductor–liquid heterojunction interface, which was largely responsible for the effective separation of photogenerated electron–hole pairs that provided the outstanding performance of our photodetector.

## 1. Introduction

Ultraviolet (UV) radiation with wavelengths from 200 to 280 nm is absorbed by the ozone layer and does not reach the surface of the Earth; this is known as solar-blind ultraviolet light. Photodetectors that operate within this wavelength range are termed solar-blind photodetectors, which offer the benefits of a high signal-to-noise ratio (SNR), low false alarm rates, the capability for all-weather operation, and immunity to the interference of sunlight. Solar-blind photodetectors offer many promising applications, including solar-blind imaging, ultraviolet optical communication, monitoring of the ozone layer, forest fire alarms, and general ultraviolet monitoring. Wide-bandgap semiconductors are commonly utilized in the fabrication of solar-blind ultraviolet photodetectors, such as GaN [1,2] and SiC [3], which often require the use of optical filters or doping to control the bandgap. Gallium oxide (Ga_2_O_3_) is a wide-bandgap semiconductor, with a bandgap of approximately 5 eV, and exists in five phases: α, β, γ, δ, and ϵ. At present, α- and β-Ga_2_O_3_ are commonly used to construct solar-blind photodetectors [4,5]. To date, the vast majority of photodetectors based on gallium oxide (Ga_2_O_3_) have employed the metal–semiconductor–metal (MSM) configuration, which mostly functions with an external power source. This limitation restricts the application of solar-blind photodetectors in a variety of environments, while simultaneously posing a potential environmental hazard [6,7], which increases the demand for self-powered photodetectors. PN junctions, Schottky barriers, and photoelectrochemical detectors can all achieve self-powered detection capabilities. Among these devices, PEC photodetectors are gaining attention for their low energy consumption, which allows them to operate self-sufficiently without the need for external power sources. This feature not only minimizes the environmental impact but also reduces the initial investments and long-term operational costs, thus contributing to sustainable development. Through these features, PEC UV detectors not only demonstrate high performance and advanced capabilities technically but also offer significant advantages economically and environmentally. PEC-type solar-blind UV photodetectors based on Ga_2_O_3_ demonstrate a remarkable photoresponse under UV radiation [5,8,9,10]. For PEC-type detectors, the electrolyte plays a pivotal role in facilitating pure ionic conductivity between the counter electrode and the photoanode. To ensure the functionality of PEC-type ultraviolet (UV) detectors, several criteria must be met: (a) The electrolyte should be capable of efficiently transporting electrons and holes between the photoanode and the counter electrode. Photogenerated holes should be swiftly oxidized by electron donors present in the electrolyte. (b) The electrolyte must promote the rapid migration of charge carriers and maintain a substantial contact area with both the photoanode and the counter electrode. (c) It is essential that the electrolyte exhibits chemical stability and remains inert to the materials of the photoanode to ensure the reliable operation and enduring stability of the PEC system. Electrolytes can be differentiated based on their physical state, their chemical composition, and the mechanisms of their formation into categories such as aqueous [5], organic liquid [11], and solid-state gel electrolytes [12]. Due to their safety, stability, and environmental benignity, aqueous electrolytes are particularly well suited for PEC UV detectors. Nevertheless, research on the influence of electrolytes on PEC detectors is quite limited. In this work, PEC detectors that utilized a variety of electrolytes were fabricated to explore the effect on the performance of the photodetector.

## 2. Materials and Methods

**Figure 1:**
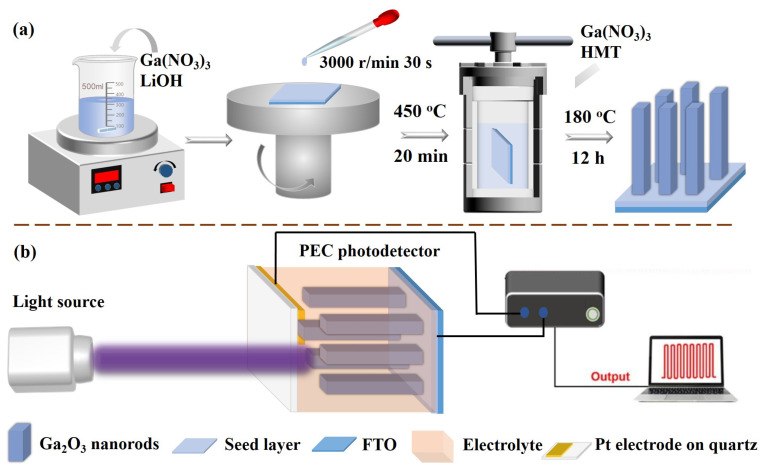
(**a**) The schematic diagram of the processing of the α-Ga_2_O_3_ nanorods. (**b**) The measurement setup of the α-Ga_2_O_3_-nanorod PEC photodetector.

The process of preparing the gallium-oxide-based PEC photodetector involved several steps. Initially, a gallium oxide sol–gel solution (50 μL) was prepared; it consisted of ethanol solution with Ga(NO_3_)_3_ and LiOH. This mixture was applied to a fluorine-doped tin oxide (FTO) substrate via spin-coating at a rotation speed of 3000 rpm for a duration of 30 s. The coated substrate was then subjected to an annealing process in a furnace preheated to 450 °C for 20 min, resulting in a uniform Ga_2_O_3_ seed layer. Subsequently, the annealed FTO substrate was immersed into a 30 mL precursor solution with a concentration of 0.03 M Ga(NO_3_)_3_. It underwent a hydrothermal reaction at 180 °C for 720 min to obtain GaOOH. After the reaction, the samples were rinsed and dried. The final transformation of GaOOH to α-Ga_2_O_3_ was achieved through annealing in a muffle furnace under an atmospheric environment, as illustrated in Figure 1a. The fabrication of the PEC UV detector was a three-step procedure: (1) α-Ga_2_O_3_ nanorods on the FTO substrate acted as the photoanode; (2) a high-transmittance quartz plate, which was partially coated with Pt to facilitate current conduction and reception of solar-blind UV signals, was employed as the counter electrode; and (3) different 0.1 M aqueous solutions, namely, Na_2_CO_3_, Na_2_SO_4_, NaOH, and pure water, were used as the electrolytes. The photoanode and counter electrodes were securely bonded using a 60 µm thick Bynel film, and the electrolyte was injected through a strategically placed hole on the counter electrode.

The morphology of the α-Ga_2_O_3_ nanorods was examined through field-emission scanning electron microscopy (FE-SEM, TESCAN, Brno, Czech Republic) and transmission electron microscopy (TEM, JEOLJEM-2100, JEOL, Tokyo, Japan). The crystallographic properties of α-Ga_2_O_3_ nanorods were characterized using X-ray diffraction (XRD, PANalytical Empyrean, Cu Ka Radiation, Alemlo, Poland). The chemical composition and valence states of the α-Ga_2_O_3_ nanorods were elucidated using X-ray photoelectron spectroscopy (XPS, Thermo K-Alpha, Thermo Fisher(CN), Shanghai, China). The performance testing of the PEC solar-blind UV detectors was conducted as illustrated in Figure 1b. The responsivity test of the devices was carried out by a 500 W xenon light source with a full-automatic grating monochromator to split the light, with a calibrated standard single crystal silicon solar cell (S1337-1010BQ, Hamamatsu, Shizuoka, Japan) as the reference cell, and current–voltage curves were collected by a Semiconductor Parametric Analyzer (FS-Pro, Primarius, Shanghai, China).

## 3. Results and Discussion

**Figure 2:**
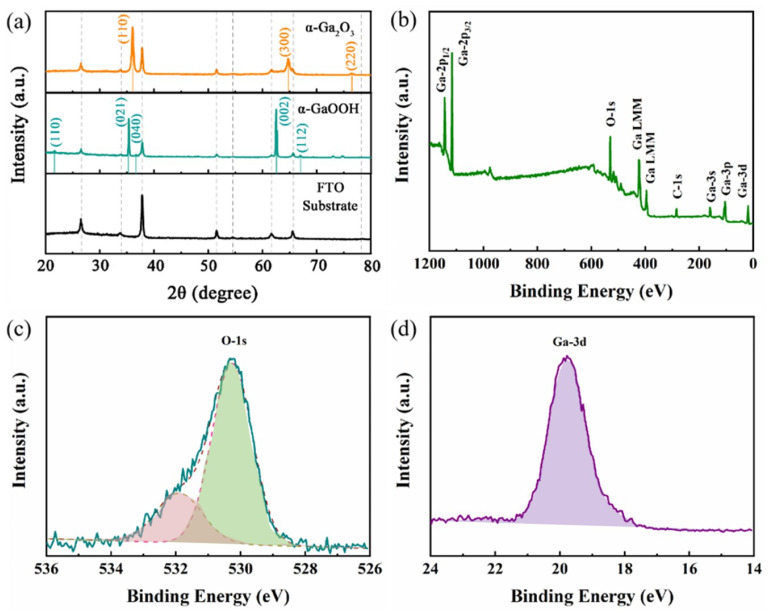
(**a**) X-ray diffraction (XRD) patterns of the prepared α-Ga_2_O_3_, the intermediate product GaOOH, and the FTO substrate. XPS patterns of α-Ga_2_O_3_ nanorods array: (**b**) survey spectrum, (**c**) O 1s, and (**d**) Ga 3d.

Figure 2a shows the XRD patterns of the FTO substrate, intermediate product GaOOH, and α-Ga_2_O_3_ nanorods. Regarding the intermediate product GaOOH, five distinctive peaks were observed at approximately 21.5°, 35.3°, 36.6°, 62.4°, and 66.8°. The distinct peaks corresponded to the (110), (021), (040), (002), and (112) planes of α-GaOOH, as indexed with the JCPDS card no. 54-0910. The noticeable shift in the positions of the XRD peaks confirmed that a phase transformation from α-GaOOH to α-Ga_2_O_3_ indeed occurred during the annealing process. The XRD pattern of α-Ga_2_O_3_ displayed a primary sharp peak at approximately 36.0°, which was complemented by two subsidiary peaks at 64.7° and 76.4°. These peaks were associated with the (110), (300), and (220) planes of the trigonal system of α-Ga_2_O_3_ (JCPDS card no. 06-0503), respectively. This observation suggests that the synthesized α-Ga_2_O_3_ exhibited superior crystallinity and predominantly grew in a direction perpendicular to the (110) plane [13]. The X-ray photoelectron spectroscopy (XPS) patterns of the as-grown α-Ga_2_O_3_ are presented in Figure 2b–d. The survey pattern of α-Ga_2_O_3_, as depicted in Figure 2b, reveals the presence of only gallium (Ga) and oxygen (O), thereby confirming the high purity of the as-grown α-Ga_2_O_3_. The principle peak in Figure 2c can be attributed to the Ga 3d from α-Ga_2_O_3_ at a binding energy of 20.2 eV [14]. The O 1s core level spectrum of α-Ga_2_O_3_, as shown in Figure 2d, was deconvoluted into two distinct components. The dominant peak, observed at 530.8 eV, is characteristic of Ga–O bonds, and signified the fully oxidized state of gallium within the α-Ga_2_O_3_ structure. This peak was a clear indication of the material’s intrinsic bonding configuration. The minor shoulder at 532.1 eV was likely associated with ionization from weakly adsorbed surface species, which may have arose from interactions with the ambient atmosphere [15,16].

**Figure 3:**
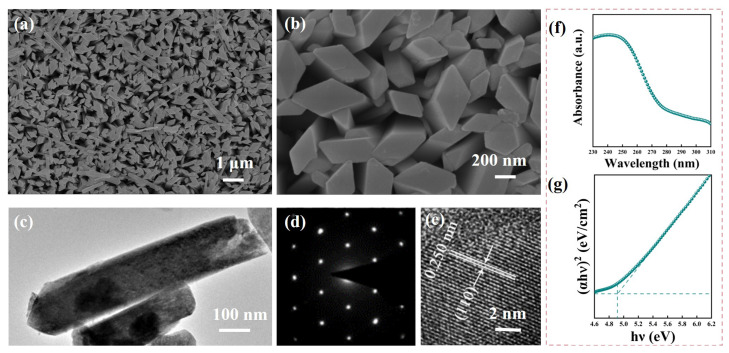
(**a**) Top view of the SEM image of the α-Ga_2_O_3_ nanorod array. (**b**) A high-magnification view of the SEM image from (**a**). (**c**) TEM image of an α-Ga_2_O_3_ nanorod. (**d**) SAED pattern of an α-Ga_2_O_3_ nanorod. (**e**) High-resolution TEM image of an α-Ga_2_O_3_ nanorod. (**f**) Absorptance spectrum of an α-Ga_2_O_3_ nanorod. (**g**) Tauc plot of an α-Ga_2_O_3_ nanorod array.

The scanning electron microscopy (SEM) images clearly show an array of α-Ga_2_O_3_ nanorods on the FTO substrate, as depicted in Figure 3a and the magnified view in Figure 3b. It is evident that the α-Ga_2_O_3_ nanorods grew vertically on the FTO substrate with a high density. Each nanorod had a characteristic rhombohedral cross-section, with an average side length that varied from 200 to 300 nm, as shown in Figure 3b. The transmission electron microscopy (TEM) image further elucidates the structure of the α-Ga_2_O_3_ nanorods in Figure 3c. The rough surface of the nanorods was likely due to the variations in density that occurred during the dehydration process when converting from α-GaOOH to α-Ga_2_O_3_. The selected area electron diffraction (SAED) pattern shown in Figure 3d confirmed that the prepared α-Ga_2_O_3_ nanorods were indeed single crystals. The high-resolution transmission electron microscopy (HR-TEM) image, as shown in Figure 3e, revealed a lattice spacing of 0.250 nm, which corresponded to the (110) plane of the trigonal system of α-Ga_2_O_3_ [13]. Figure 3f demonstrates that the α-Ga_2_O_3_ nanorod array exhibited significant light absorption in the ultraviolet region, which reached a maximum at 250 nm. To determine the optical bandgap of the α-Ga_2_O_3_ nanorod array, the following equation could be employed:

(1) $${\left(\alpha h\nu \right)}^{2}=A\left(h\nu -Eg\right),$$

where α, hν, and *A* in this equation present the optical absorption coefficient, photon energy, and constant, respectively. The optical bandgap (Eg) of the α-Ga_2_O_3_ nanorod array was determined through a methodical approach that involved the linear extrapolation to the *h*ν-axis. The graphical representation of this analysis is captured in Figure 3g, where the curve of (α*h*ν${)}^{2}$ is plotted against hν. By applying this technique, the bandgap of the α-Ga_2_O_3_ nanorod array was deduced to be approximately 4.93 eV.

**Figure 4:**
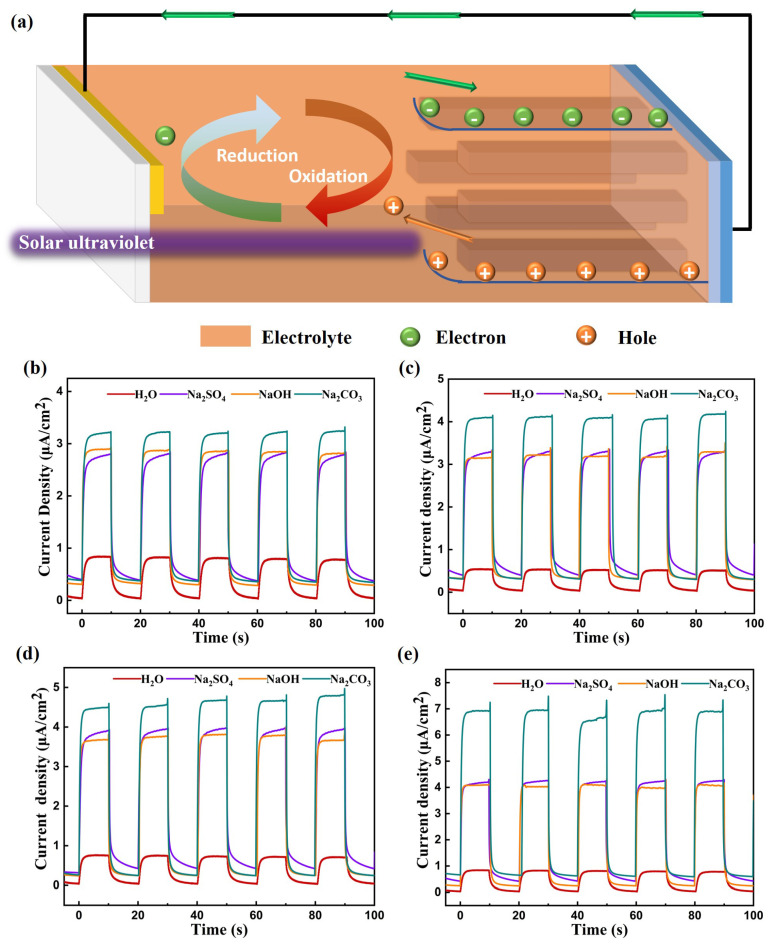
(**a**) The schematic diagram of the working mechanism of the PEC photodetector. The I–t curves of the PEC photodetector with different electrolytes under (**b**) 2 mW/cm^2^ irradiation, (**c**) 3 mW/cm^2^ irradiation, (**d**) 4 mW/cm^2^ irradiation, and (**e**) 5 mW/cm^2^ irradiation.

In a PEC photodetector, the presence of the redox couple in the solution alters the distribution of charge carriers within α-Ga_2_O_3_, creating a space charge region and inducing upward band bending [17]. When ultraviolet light with wavelengths shorter than 300 nm traverses high-transparency quartz and is absorbed by α-Ga_2_O_3_ nanorods, electron–hole pairs are produced by photons with energies that exceed the bandgap of α-Ga_2_O_3_. The built-in electric field at the interface of α-Ga_2_O_3_ and the electrolyte acts as a driving force to separate these photogenerated electron–hole pairs [15,18,19]. The separated electrons are collected by FTO along the α-Ga_2_O_3_ nanorods and are channeled into the external circuit, while the photogenerated holes migrate toward the α-Ga_2_O_3_ electrolyte interface. Here, the holes ($h^{+}$) that reach the interface are captured by active ions from the electrolyte and engage in a redox reaction to form the redox molecule. The redox molecule, which is propelled by a concentration gradient, migrates to the surface of the Pt electrode, where it recombines with electrons ($e^{-}$) that have re-entered from the external circuit, and then undergoes another redox reaction before being reduced back to ions, as shown in Equations (2) and (3) [20,21]. This sequence of reactions completes the circuit, enabling the PEC device to operate in a self-powered mode [22,23], as depicted in Figure 4a.

(2) $$h^{+}+{\mathrm{OH}}^{-}\to {\mathrm{OH}}^{*},$$

(3) $$e^{-}+{\mathrm{OH}}^{*}\to {\mathrm{OH}}^{-},$$

Electrolytes can be classified into categories that include aqueous electrolytes, organic liquid electrolytes, and solid-state gel electrolytes. Aqueous electrolyte attributes mean they are considered as one of the safest, most stable, and eco-friendly electrolytes, and thus, are deemed more appropriate for PEC UV detectors. This study conducted a comparative analysis of the photoelectric detection capabilities of α-Ga_2_O_3_-based PEC detectors using different aqueous solutions as electrolytes. Furthermore, it explored the influence of various electrolytes on the surface morphology of α-Ga_2_O_3_ nanoarrays.

PEC-type ultraviolet photodetectors were fabricated using different electrolytes, including a 0.1 M solution of the strong alkali sodium hydroxide (NaOH), a 0.1 M solution of sodium carbonate (Na_2_CO_3_), and a 0.1 M solution of sodium sulfate (Na_2_SO_4_) as electrolytes, with pure water (H_2_O) as the control electrolyte. The performances of these detectors were then characterized by I–t curves. Figure 4b–e illustrate the I–t curves for the detectors prepared with electrolytes of identical concentration but varying compositions under exposure to 254 nm ultraviolet light at power densities ranging from 2 to 5 mW/cm^2^.

Notably, regardless of the light power density, the photocurrent generated by devices that employ ionic solutions as electrolytes significantly outperformed that of devices with pure water (H_2_O) as the electrolyte. This enhanced photocurrent was likely due to the high concentration of mobile ions in the ionic solutions, which not only boosted the electrolyte’s conductivity but also expedited the separation of electron–hole pairs, thereby enhancing the overall efficiency of the photoelectric conversion process. Figure 4b–e indicate that regardless of the type of electrolyte used in the photodetectors, the photocurrent increased as the light power increased. In particular, photodetectors with Na_2_CO_3_ as the electrolyte exhibited a significantly elevated photocurrent compared with those that used other electrolytes. Conversely, there was little to no significant difference in the photocurrent between detectors that employed NaOH or Na_2_SO_4_ as the electrolyte. Sodium hydroxide (NaOH), which is renowned for its strong alkalinity, is a commonly used electrolyte solution, while sodium carbonate (Na_2_CO_3_) is a salt formed from a strong base and a weak acid. They are both adept at providing a generous amount of hydroxide ions (OH^−^), which are crucial for promoting vigorous redox reactions that involve electrons and holes. Considering the corrosive impact that strong acids and alkalis can have on metal oxide materials, it is important to recognize that these substances can intensify surface imperfections. This, in turn, can lead to a degradation in the performance and sensitivity of photoelectrochemical (PEC) ultraviolet detectors.

**Figure 5:**
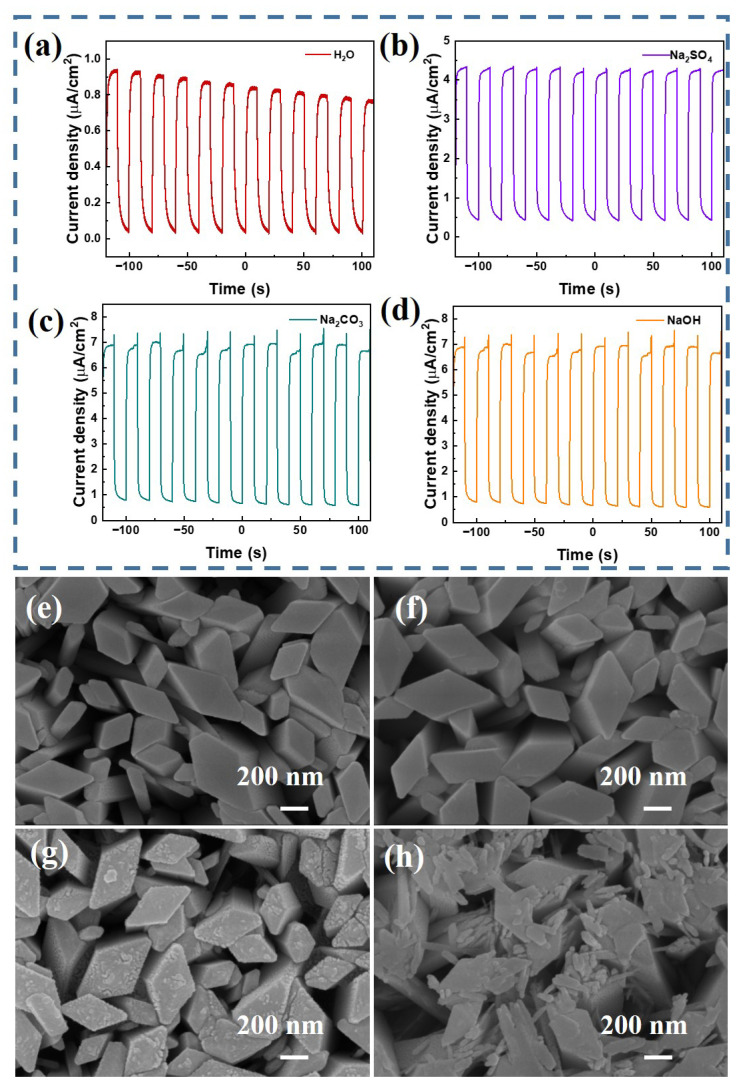
(**a**–**d**) Long-term I–t curves of the PEC detector with H_2_O, Na_2_SO_4_, Na_2_CO_3_, and NaOH as the electrolyte, respectively. (**e**–**h**) The SEM images of nanorods with H_2_O, Na_2_SO_4_, Na_2_CO_3_, and NaOH as the electrolyte, respectively.

To illustrate the impact of the different electrolytes on the performance of the detectors, long-term irradiation and I–t tests were conducted on the detectors with various electrolytes. Figure 5a reveals that the photodetectors that employed H_2_O as the electrolyte exhibited a gradual decline in photocurrent as the duration of testing time increased. Conversely, the photodetectors with electrolytes that initially demonstrated higher photocurrents, such as Na_2_CO_3_ and NaOH, experienced fluctuations in the photocurrent, suggesting a propensity for instability. However, the detectors with the Na_2_SO_4_ electrolyte displayed a very stable photocurrent. To further analyze the reasons for these phenomena, detectors were prepared with a 0.1 M Na_2_SO_4_ solution, Na_2_CO_3_ solution, or NaOH solution as electrolytes. After the electrolytes were injected, the devices were left to stand for 3 h before being disassembled. The SEM images obtained are shown in Figure 5, where Figure 5e is the blank sample without electrolyte injection, and Figure 5f–h correspond to the samples extracted from the devices after 3 h of exposure to the Na_2_SO_4_, Na_2_CO_3_, and NaOH solutions, respectively.

From the SEM images in Figure 5e,f, the Na_2_SO_4_ electrolyte had no effect on the morphology of the α-Ga_2_O_3_ nanoarrays. There were small particles on the top of the nanorods after 3 h of the Na_2_CO_3_ treament, as shown in Figure 5g. Much more particles and small nanorods were observed on the top of the α-Ga_2_O_3_ nanoarrays for the NaOH-electrolyte-treated sample, as depicted in Figure 5h, which is the reason for the unstable photocurrent of those detectors. The changes on the surface of the α-Ga_2_O_3_ nanoarrays also modulated the transport of charge carriers at the interface between the α-Ga_2_O_3_ nanoarrays and the electrolyte, which affected the redox reactions, as illustrated in Equations (2) and (3). Taking into account the previous analysis, due to the fact that the Na_2_SO_4_ electrolyte had no adverse effects on the α-Ga_2_O_3_ nanorods and the PEC detector fabricated demonstrated a stable photocurrent, the Na_2_SO_4_ aqueous solution was chosen as the optimal electrolyte for the PEC detectors.

The detailed photoelectric response performance of the PEC photodetector using Na_2_SO_4_ as the electrolyte was analyzed. The responsivity (R) of the photodetector could be calculated by this equation:

(4) $$R=\frac{I_{ph}-I_{dark}}{P\cdot S},$$

where $I_{ph}$ and $I_{dark}$ are the photocurrent and dark current, respectively. *P* represents the irradiance power density and *S* is the active area of the photodetector.

**Figure 6:**
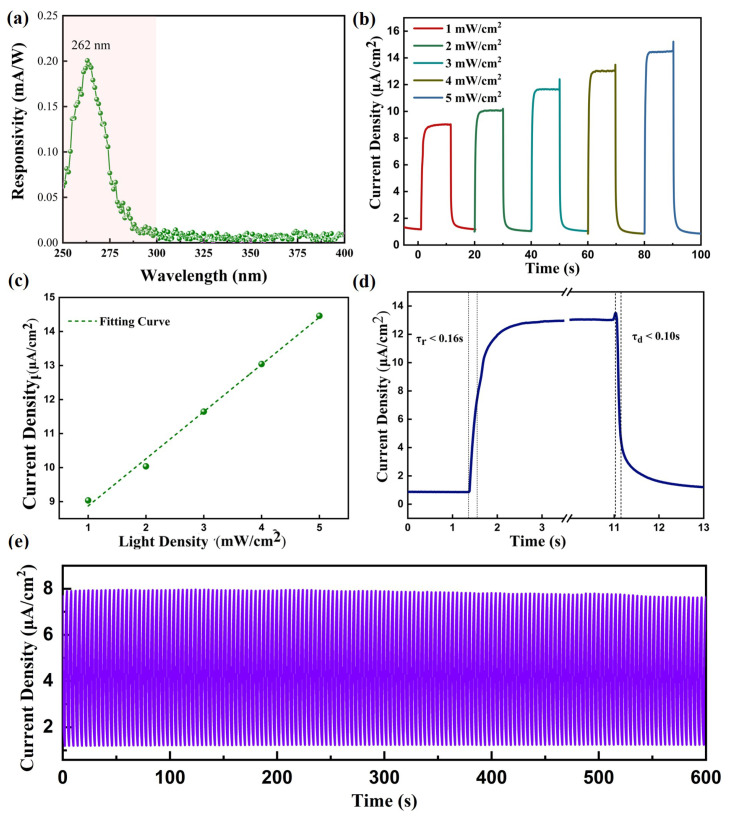
(**a**) The responsivity curve of the PEC detector with the Na_2_SO_4_ electrolyte. (**b**) I–t curves of the detector with different light powers. (**c**) The linear fit of the photocurrent density with the light power. (**d**) The response time of the PEC detector. (**e**) The stability of the PEC photodetector.

Figure 6a reveals that the peak response occurred at 262 nm with a maximum responsivity of approximately 0.2 mA/W. Figure 6b shows the I–t curve of the α-Ga_2_O_3_ PEC UV detector at zero bias as the light power density increased from 1 mW/cm^2^ to 5 mW/cm^2^. It can be seen that as the light power increased, the photocurrent density of the detector also increased. A linear fit for the photo power density–photocurrent density relationship at zero bias is shown in Figure 6c, indicating that the device exhibited excellent linear detection characteristics for solar-blind UV light signals, thereby showing it was capable of accurately measuring the magnitude of solar-blind UV light power, which is of significant practical importance. Figure 6d presents the rise and fall times of the photocurrent for the α-Ga_2_O_3_ PEC UV detector. The rise time ($\tau_{r}$) is defined as the time taken for the photocurrent to increase from its minimum to 1−1/e (approximately 63.2%) of its maximum value, with $\tau_{r}$ being less than 0.16 s. The fall time ($\tau_{d}$) is defined as the time taken for the photocurrent to decrease to 1/e (approximately 36.8%) of its maximum value from the peak, with $\tau_{d}$ being less than 0.10 s. It is evident that the improved PEC device possessed a rapid response speed, which meets the requirements for “sensitive detection.” Figure 6e depicts the stability of the α-Ga_2_O_3_-nanorod-based PEC UV detector with a Na_2_SO_4_ electrolyte under illumination with 262 nm UV light at a power of 3 mW/cm², without the application of an external bias. It was observed that under rapid switching conditions, the detector could still respond quickly to UV light, and after more than 120 cycles of repetition, the photocurrent density showed almost no fluctuation or degradation. This once again confirmed the detector’s exceptional stability and repeatability, thus allowing for long-term reuse.

For a more comprehensive comparison, the performance parameters of the PEC photodetectors presented in this paper, as well as those of recent PEC photodetectors based on α-Ga_2_O_3_, are listed in Table 1. The results not only show a good performance but also highlight the advantages, particularly in terms of the response time and the ratio of the photocurrent to dark current. The PEC detectors based on α-Ga_2_O_3_ with an appropriate electrolyte could achieve outstanding detection capabilities in the solar-blind ultraviolet spectrum.

## 4. Conclusions

This research investigated the impact of various electrolytes on the photoelectric detection capabilities of α-Ga_2_O_3_ nanorods. The I–t curves demonstrated that the PEC detector with Na_2_CO_3_ electrolyte initially exhibited higher photocurrent levels. However, as the test duration increased, the photocurrent became unstable and experienced a gradual decline. A similar trend was observed with the PEC detector with the NaOH electrolyte. In contrast, the detector with the Na_2_SO_4_ electrolyte maintained consistent detection capabilities throughout the test. The SEM characterization of samples from the disassembly of the PEC detectors revealed that the morphology of the α-Ga_2_O_3_ nanorods was significantly altered due to the effects of Na_2_CO_3_ and NaOH, which was likely a consequence of the corrosive action of the highly alkaline solution or the strong base–weak acid salt, which led to fluctuations in the photocurrent. After a comprehensive evaluation, sodium sulfate was chosen as the electrolyte for the PEC detectors and the response performance of the PEC detector was investigated. The photodetector with Na_2_SO_4_ as the electrolyte showed a maximum responsivity of 0.2 mA/W and at 262 nm with a light intensity of 3.0 mW/cm^2^, and it exhibited rise and decay times of 0.16 s and 0.10 s, respectively.

## Figures and Tables

**Table 1:** Comparison of the parameters for the Ga_2_O_3_ photodetectors reported for PEC detectors.

Structure of G$a_{2}$O_3_	Responsivity (mA/W)	Rise/Decay Time (s)	$I_{\mathrm{dark}}$/$I_{\mathrm{ph}}$	Ref.
Nanorod array	0.212	0.076, 0.056	33.74	Ref. [5]
Nanorod array	3.87	0.23, 0.15	12.81	Ref. [24]
Amorphous films on 3D CFP	12.90	0.15, 0.13	-	Ref. [25]
Nanorod array	-	<0.5, <0.2	28.97	Ref. [26]
Nanorod array	101.5	1, 3.2	-	Ref. [27]
Nanorod array	0.2	0.16, 0.10	35	This work

## Data Availability

The raw data supporting the conclusions of this article will be made available by the authors on request.
